# PCNL in COPD patient in the sit position under local infiltration anesthesia case report

**DOI:** 10.1186/s12894-020-00640-3

**Published:** 2020-06-17

**Authors:** Jianpo Zhai, Hai Wang, Xiao Xu, Zhenhua Liu, Libo Man

**Affiliations:** Department of Urology, Beijing Jishuitan Hospital, No68.Huinanbei Road, Changping District, Beijing, 100096 China

**Keywords:** Percutaneous Nephrolithotomy, Sit position, Local anesthesia, Renal calculi, Case report

## Abstract

**Background:**

Percutaneous nephrolithotomy is traditionally performed in the prone or supine position. We report the first case of percutaneous nephrolithotomy in sit position under local infiltration anesthesia.

**Case summary:**

A 69-year-old male presented with left flank pain. Kidney B ultrasound and computed tomography scan showed multiple left renal calculi and hydronephrosis. He had a long history of chronic obstructive pulmonary disease, with severe ventilatory and cardiac dysfunction, and cannot tolerate the prone or supine position. The patient received the surgery in sit position under local infiltration anesthesia. The operative time was 1 h. The visual analogue scale score during the surgery was 3. The patient had no intraoperative and postoperative complications. The postoperative plain radiography showed no residual stone fragments.

**Conclusions:**

We believe that in high-risk patients who need to undergo PCNL, a combination of sit position and local infiltration anesthesia is an alternative method.

## Background

Percutaneous nephrolithotomy (PCNL) is the standard treatment for large and/or complex renal calculi [1]. It is traditionally performed in the prone position, as it provides wider space for percutaneous puncture, instrument manipulation, and additionally, most urologists are familiar with it [2]. Various different positions for PCNL have emerged in recent decades. These include supine, lateral and modified supine position [3, 4]. In supine position, the operative time of PCNL was decreased, as there is no need for repositioning and the anaesthetist can access to the airway quickly. In the lateral position, the cardiac and respiratory parameters of patients are improved and can be easily controlled. However, PCNL in sit position has not been reported. The site position can decrease the cardiac preload and increase the ventilation, thus it has the minimal impact on the respiratory and circulatory functions. We report the first case of PCNL in sit position under local infiltration anesthesia.

## Case presentation

**Fig. 1:**
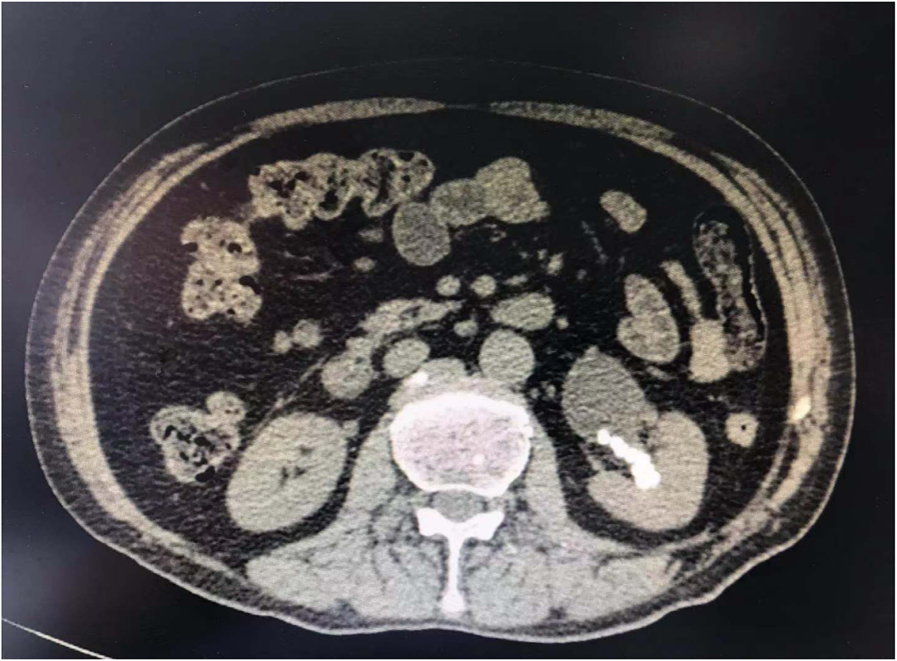
CT scan showed left renal calculi and hydronephrosis

The patient was a 69-year-old male, complaining of intermittent left flank pain for 2 years. The pain recurred every 2-3 months, without nausea and vomiting, and could be relieved by the analgesic drug. One month before hospitalization the frequency of severe flank pain steadily increased. Kidney B ultrasound and CT scan showed multiple left renal calculi and hydronephrosis (Fig. 1). There were approximately 20 stones of variable size in the left renal calyx and pelvis, with the maximum diameters of 1 cm. He had a previous medical history of chronic obstructive pulmonary disease (COPD) for 10 years and had smoked for 50 years (2 cigarettes a day and withdraw for 1 year). He usually had the symptoms of cough and shortness of breath. His pulmonary function test showed a severe ventilatory dysfunction with FEV1/FVC<70, 30%<FEV1<50% predictive value. Echocardiography showed that the left ventricular ejection fraction of the patient was 55%. ASA score levels III.

The patient received PCNL under local anesthesia in site position. Lornoxicam (8 mg) premedication was applied half an hour before surgery. Ureteral catheterization was performed under 1% lidocaine urethral surface anesthesia, and then a Foley catheter was placed into the bladder with the ureteral catheter fixed to it.

**Fig. 2:**
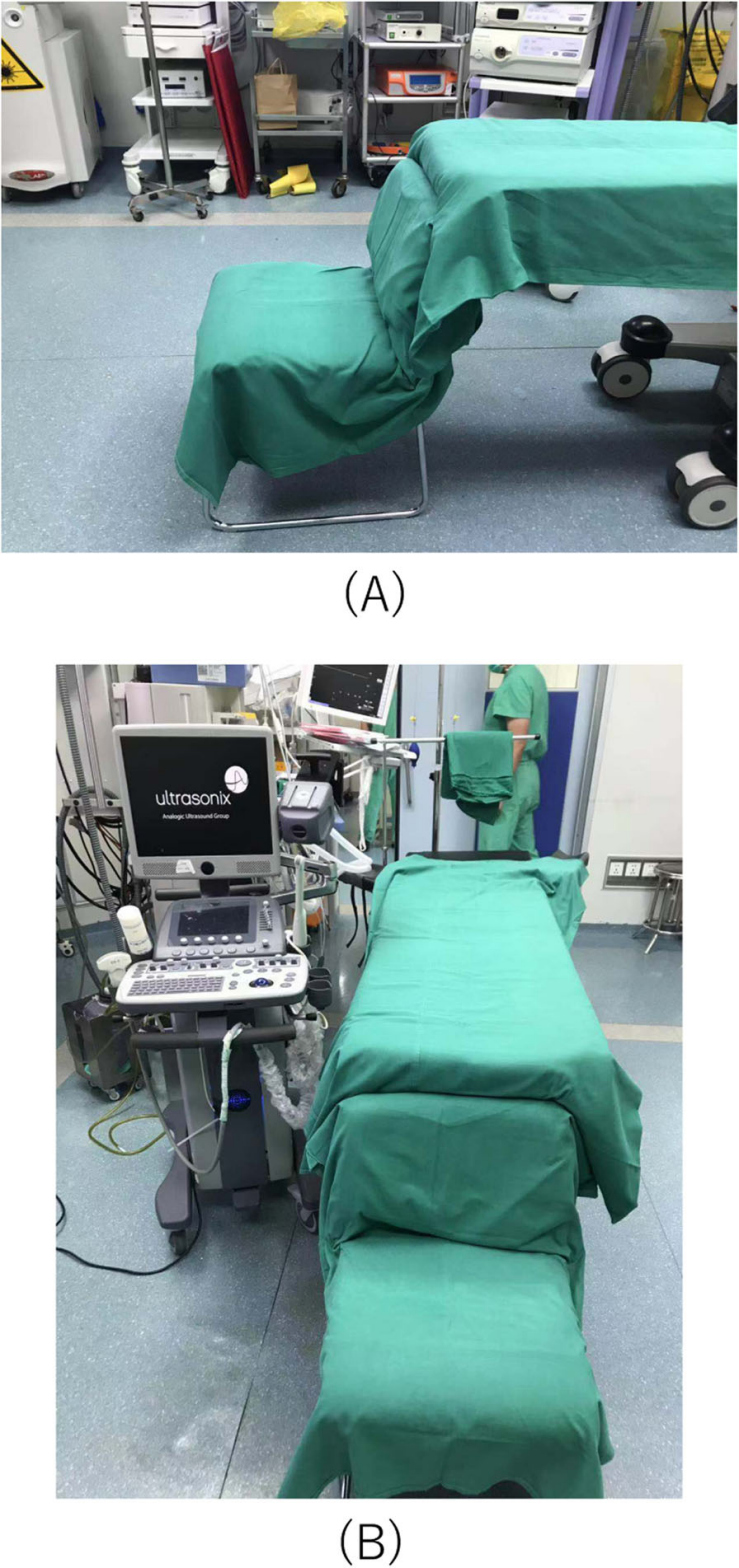
**a** Chair with back was put at the rear end of the operating table. **b** The Ultrasonic instrument was placed on the left side of the chair

**Fig. 3:**
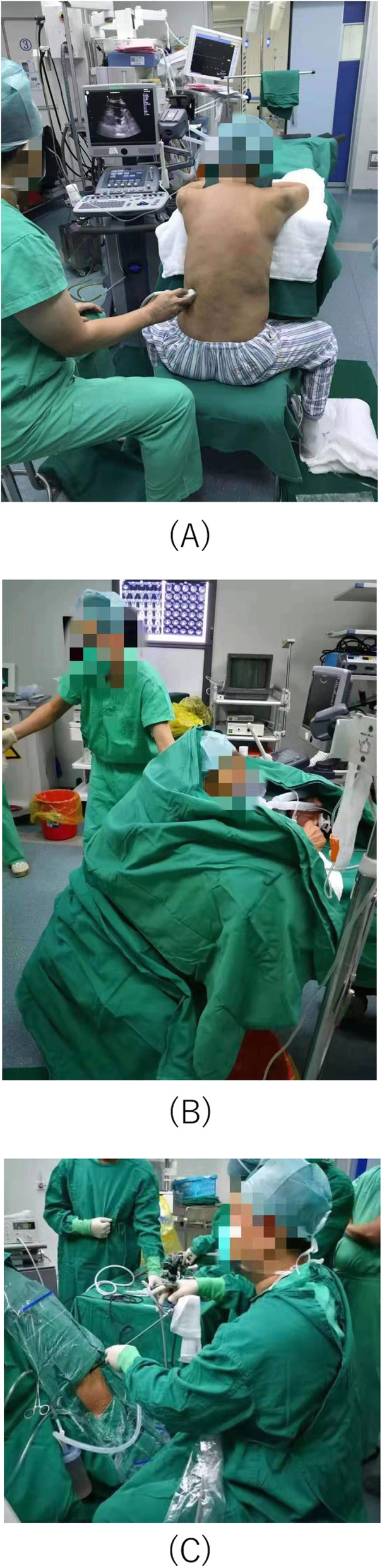
**a** The patient reversely rode on the chair with arms crossed on the operating table. **b** The skin of surgical area was cleaned with iodine and alcohol, then sterile drapes were applied to cover all areas except the surgical field. **c** The urologist performed the surgery in the sit position

A chair with back was put at the rear end of the operating table (Fig. 2). The patient reversely rode on the chair with arms crossed on the operating table. The Ultrasonic instrument was placed on the left side of the patient with the urologist sitting on the same side. The skin of surgical area was cleaned with iodine and alcohol, then sterile drapes were applied to cover all areas except the surgical field (Fig. 3). The electrocardiography and oxygen saturation were monitored during all procedures.

**Fig. 4:**
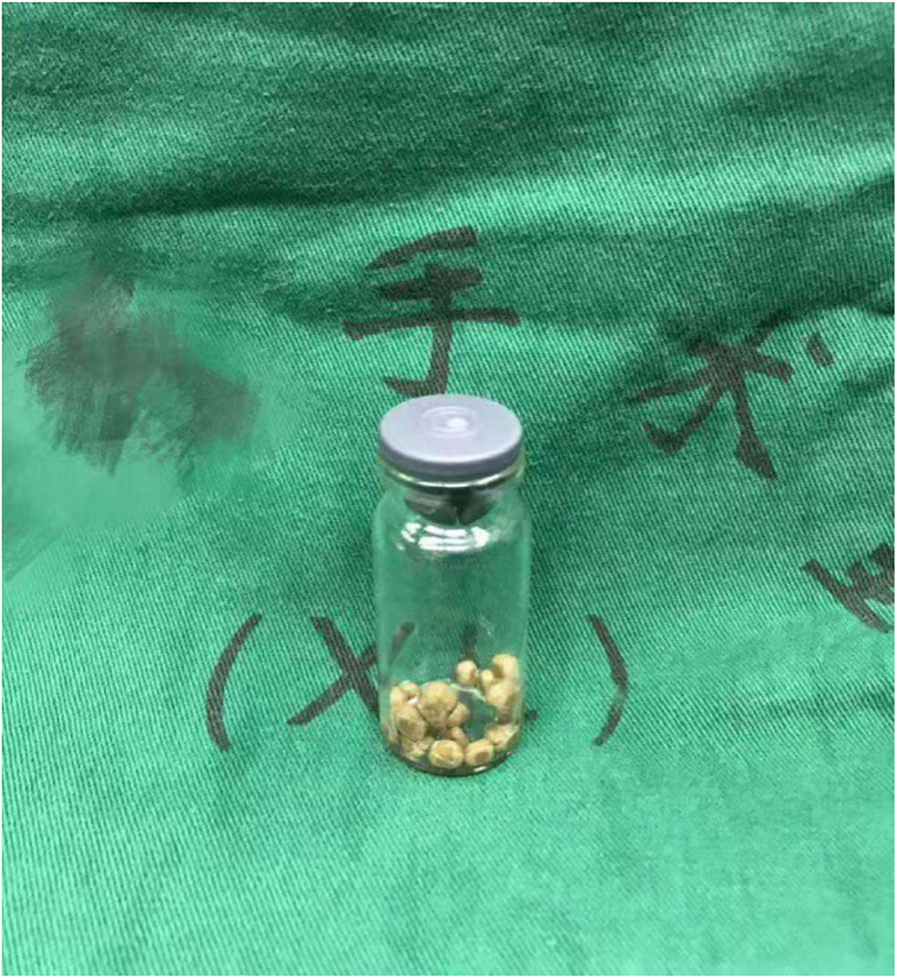
Stone fragments taken out through the PCNL access

PCNL was performed under lidocaine hydrochloride infiltration anesthesia. The puncture site was determined by the ultrasonography after observation of the calculi. The 12th subcostal space between the posterior axillary line and scapular line was chosen as the puncture site. Lidocaine (1%) was infiltrated the skin, subcutaneous tissue, muscles, renal capsule, and the underlying parenchyma by the use of a 23-gauge spinal needle. The total usage of lidocaine was 15 mL. An 18-gauge coaxial nephrostomy punction needle was made into the middle calyx under the guidance of ultrasonography. After the effluxion of urine, a J-guidewire was inserted through the punction needle into the collecting system. A 1-cm skin incision was made, and a 18F working sheath was placed as the percutaneous tract. The stone was fragmented by pneumatic lithotripsy system under the direct vision of ureteroscope (8/9.8F). The stone fragments were taken out by grasping forceps or stone basket (Fig. 4). Finally, a 6F double-J stent was inserted in the ureter and a 14F nephrostomy tube was placed in the pelvis.

The operative time was 1 h. The visual analogue scale (VAS) score during the surgery was 3. The patient had no intraoperative and postoperative complications. The postoperative plain radiography showed no residual stone fragments.

## Discussion and conclusions

PCNL is traditionally performed in the prone position. It offers a wide operative field, which can make the surgeon to puncture and dilate the tracts more easily, however it has potential disadvantages, such as it may cause circulatory and ventilatory compromise, especially in the obese or skeletal malformation patients and it is not feasible for the anterograde and retrograde access simultaneously [5, 6]. The supine PCNL position can overcome these drawbacks stated above. In addition, the operative time can be decreased, as there is no need for re-positioning and allow quick access to the airway for the anesthetist [7]. However, Patients with marginal lung and heart function can not tolerate the prone or supine position well. These problems are further compounded through general anesthesia, which uses muscle paralysis. In order to decrease the influence of PCNL on the respiratory and circulatory functions, we decided to treat him in the sit position under local anesthesia.

We reported the first case of PCNL in sit position. The patient had a long history of COPD and smoking, with severe ventilatory and cardiac dysfunction, and cannot keep in the prone or supine position for a long time. The patient received the surgery in sit position, which has several advantages. Firstly, the venous return to the heart decreased and the ventilation increased during the surgery for the patient in a comfortable sit position. Therefor the sit position, which has minimally impact on the respiratory and circulatory functions, can be used in patients with severe cardio-pulmonary dysfunction. Secondly, the surgeon can also do the surgery in the comfortable sit position, which is labor saving. Thirdly, the patient reversely rode on the chair, bend over the chairback. The intercostal space widened, which can ease the puncture and dilation. Moreover the kidney moved down when the patient maintained in the sit position, the risk of pleural injury during the surgery was decreased.

The development of PCNL under local anesthesia has several reasons, including that general anesthesia is not optional for some patients due to severe comorbidity, the need for cost suppression and hospital-stay reduction. Hulin li et al. [8] described their experience of 2000 cases of PCNL under local infiltration anesthesia. Thorsten H. Ecke et al. [9] retrospectively analyzed 439 patients of PCNL under local anesthesia and demonstrated that PCNL performed under local infiltration anesthesia was a feasible method, which can provide satisfactory positive clinical outcomes.

The pain during PCNL procedure is mainly caused by the dilatation of the renal capsule and parenchyma and not by stone fragmentation. Therefore, the renal capsule should also be infiltrated with the lidocaine [10]. We firstly used the ultrasonography to observe the position of calculi, to choose the puncture site and direction, and then injected lidocaine by the use of a 23-gauge spinal needle from the skin to the renal parenchyma, to ensure that lidocaine would be infiltrated along the entire tract. In our case report, the patient gave the VAS sore of 3, and tolerated the surgical procedure well without changing the type of anesthesia or increasing the usage of anesthetic drug.

The best indication of PCNL in sit position under local infiltration anesthesia is for patients with renal and/or upper ureter calculi that can be treated by the single-tract PCNL,and patients with severe cardiopulmonary dysfunction or skeletal malformation. As these patients are unable to maintain in the prone or supine position for a long time. Therefore we believe that in these high-risk patients who need to undergo PCNL, a combination of sit position and local infiltration anesthesia is an alternative method.

## Data Availability

The datasets used and/or analysed in the current study is available from the corresponding author on reasonable request.

## Abbreviations

PCNL: Percutaneous nephrolithotomy
COPD: Chronic obstructive pulmonary disease
